# Camembert-Type Cheese with Sweet Buttermilk: The Determination of Quality Properties and Microstructure

**DOI:** 10.3390/foods13162515

**Published:** 2024-08-12

**Authors:** Katarzyna Szkolnicka, Izabela Dmytrów, Anna Mituniewicz-Małek, Batoul Meghzili

**Affiliations:** 1 Department of Toxicology, Dairy Technology and Food Storage, Faculty of Food Sciences and Fisheries, West Pomeranian University of Technology, Papieża Pawła VI St. no. 3, 71-459 Szczecin, Poland; izabela.dmytrow@zut.edu.pl (I.D.); anna.mituniewicz-malek@zut.edu.pl (A.M.-M.); 2 Agro-Food Engineering Laboratory (GENIAAL), Institute of Nutrition, Food and Agro-Food Technologies (INATAA), University Frères Mentouri—Constantine 1 (UFMC1), Route Ain El Bey, Constantine 25000, Algeria; batoul.meghzili@yahoo.fr

**Keywords:** camembert-type cheese, sweet buttermilk, ripening, microstructure, texture

## Abstract

Camembert is a type of surface-mold-ripened soft cheese traditionally produced from cow’s milk. Buttermilk, a by-product of butter production with beneficial nutritional and technological properties, is increasingly being used in various applications, including cheesemaking. Therefore, this study aimed to use sweet buttermilk (BM) in combination with milk at concentrations of 10% (*w*/*w*) (BM10) and 20% (*w*/*w*) (BM20) for the production of Camembert-type cheese. A control cheese made entirely from milk was also produced. The cheese samples underwent a 28-day ripening process during which their composition, acidity, water activity, color, and sensory properties were examined at 1-week intervals. The microstructure of the matured Camembert-type cheese samples was analyzed using scanning electron microscopy (SEM), and their texture was evaluated. The production yield of BM20 cheese (18.03 ± 0.29 kg/100 kg) was lower (*p* < 0.05) than that of the control (19.92 ± 0.23 kg/100 kg), with BM10 showing the distinctly lowest yield (14.74 ± 0.35 kg/100 kg). The total solid and fat content of BM Camembert-type cheese samples was lower than the control. However, the total protein content in cheese BM20 at the end of the ripening period was the same as that of the control. The changes in acidity in all samples were typical for Camembert cheese, and water activity was high (above 0.92). The sensory properties of all samples were characteristic of the cheese type, while the color of BM cheese samples differed from the control. The microstructure of BM10 and BM20 cheese variants was similar, namely homogenous and less porous compared to the control. In terms of texture, the BM samples had significantly lower hardness, adhesiveness, and gumminess. This study indicates that sweet BM, particularly at a concentration of 20%, may be effectively used in the production of Camembert-type cheese.

## 1. Introduction

Camembert is a soft, high-moisture cheese in a cylindrical shape, covered with a velvety white layer of mold, typically *Penicillium camemberti*, *Penicillium candidum*, or *Geotrichum candidum*. Camembert cheese originates from France and is traditionally made from unpasteurized cow’s milk [1]. After the cheese is shaped, it is sprinkled with dry salt on the surface, and subsequently, molds are inoculated. This cheese type is characterized by a short ripening time because of high moisture content and rapid growth of surface mold within the first two weeks of ripening. The ripening process is complex, especially due to molds that have intense proteolytic activity. Ripened Camembert cheese has a bloomy edible rind, a creamy interior texture, and specific flavor characteristics. This cheese type is popular in many countries around the world [1,2,3]. Camembert cheese has a high nutritional value. It is a good source of protein and fat and contains essential nutrients such as essential amino acids and vitamins (A, E, riboflavin, niacin, B6, B12, and folate). However, the calcium level in Camembert-type cheese is relatively low in comparison with other types of ripened cheese [4]. During the ripening of Camembert cheese, as a result of β-casein hydrolysis, several bioactive peptides like ACE inhibitors, immunomodulatory agents, and anticarcinogenic peptides are released [5].

In recent years, a number of studies have been conducted on Camembert-type cheese. Galli et al. conducted a study on the peptide profile [5] and sensorial properties [6] of Camembert cheese fortified with probiotic strain *Lactobacillus rhamnosus* GG. The authors found that the addition of *Lactobacillus rhamnosus* GG improves the sensory acceptance of Camembert cheese made from pasteurized milk and enhances the production of bioactive peptides. Nam et al. [7] examined Camembert cheese with the addition of 1, 3, and 5% rice powder during the 4-week ripening period. Rice powder modified the texture of cheese and resulted in a milder Camembert flavor. Other scientists [8] fortified Camembert cheese with 0.05–0.2% red ginseng powder and obtained a functional product with antioxidative effects and a ripening grade similar to common Camembert. In turn, Hell et al. [9] developed Camembert cheese with the addition of two species of seaweeds (*Palmaria palmata* and *Saccharina longicruris*). The obtained Camembert-type cheese products were a source of bioactive and antioxidant compounds. Camembert cheese may be manufactured from sources other than cow’s milk. Gebreyowhans et al. [10] obtained Camembert cheese from cow’s milk, goat’s milk, and mixtures of the two. Cheese products made with a mixture of 50% cow’s milk and 50% goat’s milk exhibited particularly positive nutritional characteristics and consumer acceptability. Voblikova et al. [11] analyzed the fatty acid profile of Camembert-type cheese produced from sheep’s milk.

Buttermilk (BM) is a very abundant by-product of cream churning during butter production. According to Eurostat [12], in 2022, in the UE, 149.9 million tons of whole milk were processed, of which 29% was used for butter production, resulting in the production of 2.3 million tons of butter. Since the amounts of butter and buttermilk obtained during cream churning are almost equal [13], a similar amount of BM was produced. Equal amounts of butter and BM were also obtained in our own studies. Depending on the process of butter production, two types of BM may be distinguished: sweet BM from unfermented cream and acid BM from cream fermented with mesophilic lactic acid bacteria [14,15]. BM contains water-soluble components of cream (protein, lactose, and minerals), and its composition is similar to that of skim milk. Especially significant components are released to BM from the milk fat globule membrane (MFGM). These include polar lipids (phospholipids [PL], sphingolipids, glycerophospholipids), glycoproteins, and other minor compounds [16]. The polar lipid content in BM ranges from 0.1% to 0.2% and is about five times higher than in full-fat milk [17]. Proteins from MFGM account for 19% of all BM proteins [18]. Proteins from MFGM account for 19% of all BM proteins [18]. In addition, BM contains bioactive components such as vitamins (in particular B12 and riboflavin), essential fatty acids, and conjugated linoleic acid (CLA) [17,19]. MFGM is proven to have multiple health benefits, such as a cholesterol-lowering effect, a positive impact on the nervous system and brain functions, prevention of infections, and an anticancer effect [15,17,20,21,22]. Hence, BM is a promising raw material for functional food production. The applications of BM are also dictated by its emulsifying, water-binding, and structure-forming properties [23]. BM is currently used in yogurt production to improve water-binding capacity and texture properties [24,25,26], in functional ice cream production [27,28], in bakery products to improve crumb texture [29], and in chocolate as a substitution of skim milk powder [30]. Another area with significant potential for BM application is cheesemaking [15]. BM has been used in various cheese types in liquid [31,32,33], concentrated [34,35,36], and powdered form [15,37]. However, the drying of BM affects its functional properties [13]. The addition of BM increases the moisture content in cheese [17] and enhances the structure of low-fat cheese [16]. Borges et al. [35] developed reduced-fat cheese with concentrated buttermilk, which had similar texture and sensory properties to full-fat cheese. According to Sakkas et al. [15], sweet sheep buttermilk is suitable for the production of reduced-fat sheep milk cheese. Buttermilk has already been used in the production of quark cheese [14,32,38], fresh cheese with polymerized whey protein [23,39], and Cheddar-type cheese [33].

The available literature suggests that there are no research reports on the use of sweet buttermilk in Camembert-type cheese production. For this reason, the aim of this study was to investigate the impact of the use of sweet BM combined with milk in two ratios (10% and 20% *w*/*w*) on the production yield and quality properties of Camembert-type cheese. The quality of cheese samples was analyzed during a 28-day, two-stage ripening period. The effect of BM addition on the production yield, cheese composition, acidity, water activity, texture, color, and microstructure, was analyzed using scanning electron microscopy (SEM).

## 2. Materials and Methods

### 2.1. Materials

#### 2.1.1. Buttermilk Production

Buttermilk production was performed in a laboratory-scale churning machine from pasteurized sweet cream (30% fat). The cream was purchased from a local producer. From 10 kg of cream, we obtained approximately 5.4 kg of buttermilk.

#### 2.1.2. Camembert-Type Cheese Production

**Figure 1:**
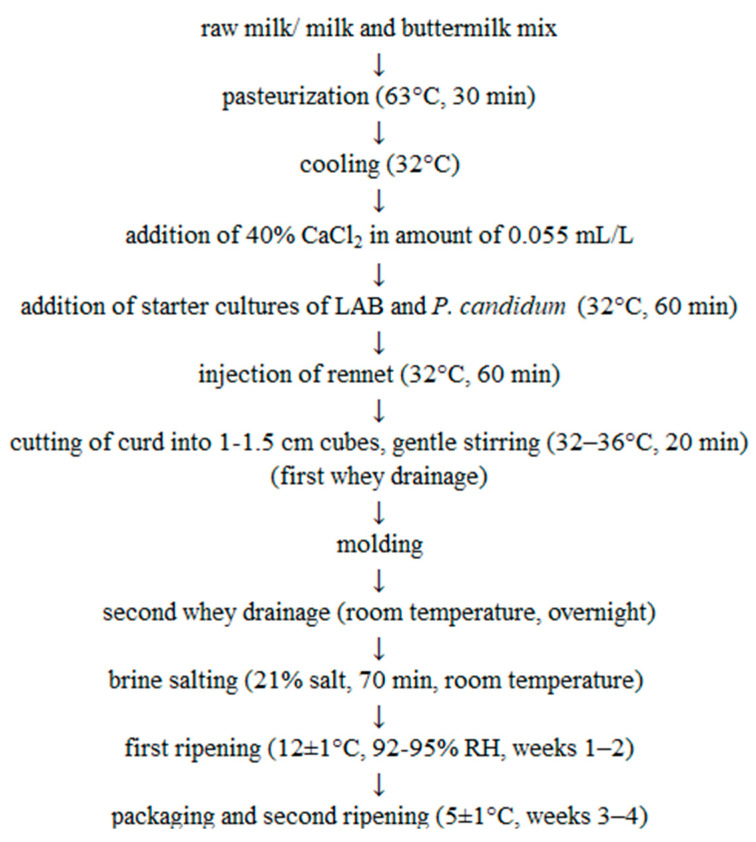
Scheme of Camembert-type cheese production [40], with modifications.

Camembert-type cheese was made from raw cow milk purchased from a local farmer and from buttermilk. Within the study, we manufactured three variants of cheese: C—control, made entirely from milk; BM10—made from milk with a 10% (*w*/*w*) addition of buttermilk; and BM20—made from milk with a 20% (*w*/*w*) addition of buttermilk. Production was carried out according to the methodology presented by Bae et al. [40], with slight modifications (Figure 1). After pasteurization and cooling of the milk or milk-and-buttermilk mixture, calcium chloride and starter cultures were added. We simultaneously added culture MST (Biochem s.r.l., Rome, Italy) containing *Lactococcus lactis* subsp. *lactis*, *Lactococcus lactis* subsp. *cremoris*, *Streptococcus salivarius* subsp. *thermophilus*, and culture PC (Biochem s.r.l., Rome, Italy) containing *Penicillium candidum*. Both cultures were in lyophilized form for direct milk inoculation. For coagulation, natural liquid rennet (Biochem s.r.l., Rome, Italy) in addition 0.2 mL/L was used. During the first 14 days of ripening, cheese discs were turned every day. After that period, all cheese discs were covered with mold. On the 14th day, each cheese disc was packaged in polyethylene-coated paper and placed in new conditions for 14 additional days. In the production process, 25 discs of each cheese variant were obtained. The mass of each disc was approximately 120 g, the diameter was approximately 9 cm, and the height was approximately 3 cm. The experiment was performed in duplication. Directly after salting, cheese discs were weighed, and the production yield was calculated by dividing the weight of cheese by the weight of milk/milk-and-buttermilk mix, and the results were presented as kilograms of cheese per 100 kg of milk/milk-and-buttermilk mix [41].

### 2.2. Methods

#### 2.2.1. Physicochemical Properties

In raw materials, i.e., raw milk and buttermilk, the following parameters were analyzed: total protein content using the Kjeldahl method, fat using the Gerber method, total solids using the drying method, and titratable acidity expressed as % of lactic acid [42]. The pH was tested with a pH meter (Elmetron, Zabrze, Poland) according to the instruction manual. To ensure accuracy, prior to the experiment, the pH meter was calibrated with buffers 4.01 and 7.01. Moreover, color parameters of milk and buttermilk were tested using the CIELAB system with a colorimeter model FRU^®^ WR-18 with 8 mm an aperture (Shenzhen Wave Optoelectronics Technology Co., Ltd., Shenzhen, China), which was calibrated with the standard white plate. The following parameters were estimated: L* (lightness); a* and b*. Additionally, the whiteness index (WI) was calculated according to the following formula [43]:

(1) $$WI=100-\sqrt{{\left(100-L\right)}^{2}\;{+\;a}^{2}\;{+\;b}^{2}}$$

In Camembert-type cheese, after 1, 7, 14, 21, and 28 days of ripening, the following quality characteristics were analyzed: total solids, fat, % of lactic acid [42], pH with a pH meter (Elmetron, Zabrze, Poland), and water activity (a_w_) (HydroLab device, Rotronic AG, Bassersdorf, Switzerland). The total protein content was analyzed at the beginning and end of ripening (day 1 and day 28) using the Kjeldahl method [42]. All the reagents used in this study were of analytical grade.

#### 2.2.2. Color Parameters and Sensory Properties

Throughout the ripening period, color and sensory properties were also analyzed. Color was measured with the same equipment as in raw materials. During the ripening period, color was measured on the cross-section of the sample (in the interior). Starting from the 14th day, when the mold on the cheese surface was developed, exterior color was also measured. Color was tested each time on two discs of Camembert-type cheese in three repetitions for each disc [44].

Before sensory evaluation, cheese discs were conditioned for 1 h at room temperature of about 20 °C and then cut into triangular pieces of approximately 10 g each, coded with random numbers, and served on a white plate. Within the analysis, the following parameters were assessed: appearance, consistency, taste, aroma, and overall acceptability. A 7-point hedonic scale was used (7 points—very good; 6—good; 5—rather good; 4—moderate; 3—rather bad; 2—bad; 1—very bad quality). The analysis was performed by six panelists (two males and four females in the 30–50 age group) trained in cheese sensory assessment during two training sessions in which commercial and laboratory-produced Camembert cheese products at different stages of ripening were tested. Each of the panelists had a separate booth with white light and was provided with water to cleanse the palate between tests [10,40].

#### 2.2.3. Microstructure

The microstructure of cheese samples was measured on the 21st day of ripening using a scanning electron microscope (SEM) (VEGA3, TESCAN, Brno, Czech Republic). The cheese samples were placed on a carbon film that did not interact with the sample, and images were taken directly. To avoid damaging the delicate structure of the samples, they were not coated with a nanometric metal layer. The high voltage value (HV) was 5 kV, and working distance was 13.60–13.86 mm. In this study, the internal microstructure of Camembert-type cheese was investigated.

#### 2.2.4. Texture

At the end of the ripening period (day 28), texture profile analysis (TPA) with the use of the texture analyzer TA.XT plus (Stable Micro System, Godalming, UK) was performed. The analysis included the assessment of hardness, adhesiveness, springiness, and cohesiveness. The conditions during the texture analysis are presented in Table 1 [7,40,44].

#### 2.2.5. Statistical Analysis

The results underwent statistical analysis using Statistica 13.1 Software (StatSoft Inc., Tulsa, OK, USA), with a significance level of *p* = 0.05. During the analysis, mean values and standard deviations were calculated. Shapiro–Wilk’s test was used to check if the distribution of variables was normal. Differences among the samples were tested by ANOVA followed by Tukey’s post hoc test. Depending on the methodology, analyses were carried out in 3–6 repetitions.

## 3. Results and Discussion

### 3.1. Raw Materials

The characteristics of milk and buttermilk used for Camembert-type cheese production and the results of one-factor ANOVA analysis are presented in Table 2. Buttermilk had a significantly higher pH than milk, but the materials did not differ in terms of titratable acidity. Buttermilk was characterized by lower fat and total solid content but higher total protein content than milk. Higher protein content indicates that it is reasonable to use buttermilk in cheese production. Analyzing the color of raw materials, we found that buttermilk had significantly lower values of all parameters. This material was less light and had a more distinct green-yellow shade than milk. According to Gassi et al. [45], the composition and characteristics of sweet BM depend on the origin of the milk used for cream production, the parameters of cream heat treatment, and the technology of butter production, especially whether it is a slow or fast process. This is the reason for the differences in sweet buttermilk properties found in the literature. Govindasamy-Lucey et al. [34] used sweet BM with 8.26% total solids, 2.66% protein, and 0.75% fat, which is distinctly lower than in our study. Morin et al. [13] produced rennet gels from BM with 9.33% total solids, 3.11% protein, and 0.48% fat, which is also lower than our findings. Additionally, El-Dardiry et al. [46] obtained sweet BM with lower total solids, fat, and protein content (9.6%, 0.67%, and 3.56%, respectively). Sweet BM with similar to our results’ total solids, fat, and lactic acid content (10.25, 1.33, and 0.14, respectively) was tested by Bahrami et al. [31]; however, the protein content (3.05%) and pH (6.63) in their study were lower than our results.

### 3.2. Production of Camembert-Type Cheese

The production yield of control Camembert-type cheese was the highest, amounting to 19.92 ± 0.23 kg/100 kg. In the case of cheese BM20, the result was slightly lower (18.03 ± 0.29 kg/100 kg). The lowest production yield was observed in cheese BM10 (14.74 ± 0.35 kg/100 kg). The results were significantly different (*p* < 0.05). Bahrami et al. [31] studied cream cheese with 0–50% sweet buttermilk addition to milk and noted that the production yield of cheese with 10% buttermilk (17.51%) was lower than that of the control (18.16%), while the production yield of cheese with 20% buttermilk was similar to the control, amounting to 18.26%. The pH of whey obtained during the first whey drainage was significantly (*p* < 0.05) higher in the control (4.72 ± 0.01) in comparison with BM10 and BM20 (4.57 ± 0.07 and 4.61 ± 0,08, respectively). A similar value of pH (4.6) of whey drained during the production of lactic curd Camembert was obtained by Batty et al. [47].

### 3.3. Physicochemical Properties

**Figure 2:**
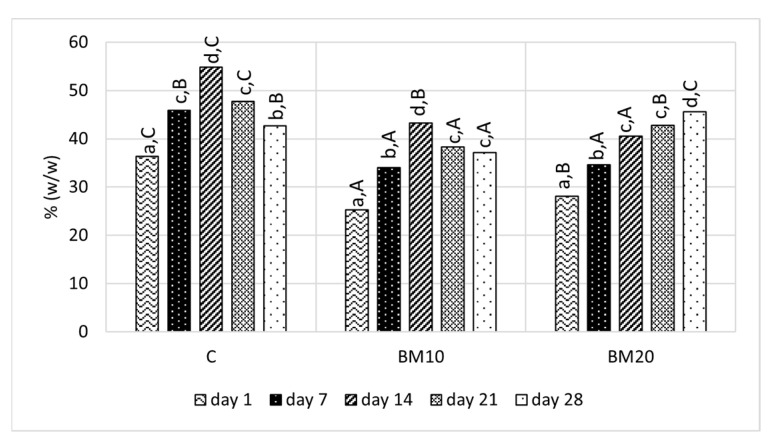
The total solid content in Camembert-type cheese during ripening. Explanatory notes: C—control Camembert-type cheese; BM10—Camembert-type cheese with 10% buttermilk; BM20—Camembert-type cheese with 20% of buttermilk. Different small letters (a–d) indicate significant differences in the same cheese on different ripening days. Different capital letters (A,B,C) indicate significant differences between cheese samples on the same ripening day (*p* ≤ 0.05).

During the first three weeks of ripening, the control cheese had a significantly higher total solid content than cheese samples with BM (Figure 2). After 4 weeks of ripening, cheese BM20 had the highest total solid content (45.64%). The lower total solid content of cheese samples with BM that was observed during most of the study period indicates the water-holding capacity of buttermilk ingredients. The presence of MFGM compounds, especially PL, provides the capacity to retain and absorb whey in the cheese structure [31]. PL and other MFGM fragments form complexes with casein and alter rennet-induced gel formation, resulting in a less dense gel structure and higher moisture content [48]. Moreover, the moisture increase in samples with BM addition could be due to the presence of denatured whey proteins. The denaturation of whey proteins may occur during cream pasteurization, and these proteins are able to form cross-links with casein micelles, which strengthens the gel and reduces the extent of syneresis [34,48]. A higher moisture content in cheese with BM addition compared to cheese without BM was also observed by Govindasamy-Lucey et al. [34], Morin et al. [13], Bahrami et al. [31], and Krebs et al. [48].

A gradual increase in the total solid content was observed during the first two weeks in both products C and BM10 when the cheese ripened without packaging. Wrapping the cheese discs with polyethylene-coated paper inhibited water loss, leading to a decrease in the total solid content during the last two weeks. However, in the case of cheese BM20, total solids continued to increase even after packaging. The total solid content in the control sample is typical for Camembert made from milk and similar to the results obtained by Batty et al. [44,47], Bensmail et al. [49], and Los et al. [50].

**Figure 3:**
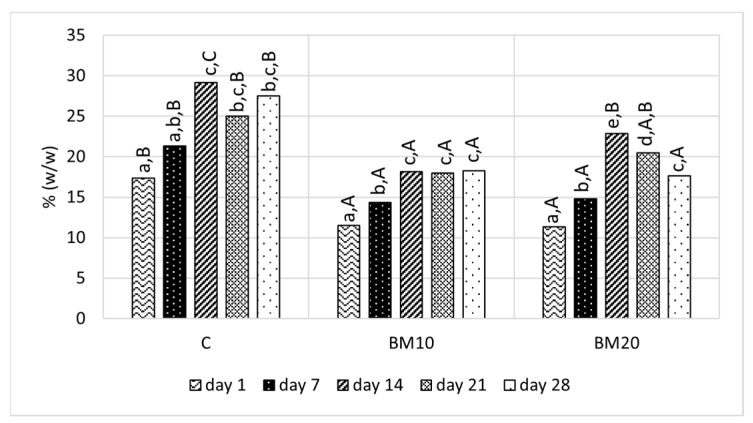
Fat content in Camembert-type cheese during ripening. Explanatory notes: C—control Camembert-type cheese; BM10—Camembert-type cheese with 10% buttermilk; BM20—Camembert-type cheese with 20% of buttermilk. Different small letters (a–e) indicate significant differences in the same cheese on different ripening days. Different capital letters (A,B,C) indicate significant differences between cheese samples on the same ripening day (*p* ≤ 0.05).

Similarly to the total solids, the lowest fat content in all cheese variants was observed on the 1st day (Figure 3). During the first two weeks, when the cheese ripened without packaging, the fat content increased. This increase is associated with the moisture loss of Camembert-type cheese [50]. Comparing the samples, it can be observed that products BM10 and BM20 had significantly lower (*p* < 0.05) fat content throughout the ripening period compared to the control, except on the 21st day, when cheese BM20 did not differ significantly from the control. This is presumably the result of lower fat content in buttermilk compared to milk (Table 2). Lower fat content in cheese with BM addition was also observed by Bahrami et al. [31]. Low levels of fat in buttermilk-enriched cheese products may be perceived as an advantage in terms of their lower calorie content. In the case of fat content in the control cheese, the results are consistent with those reported by other authors on Camembert cheese [47,49,50].

**Figure 4:**
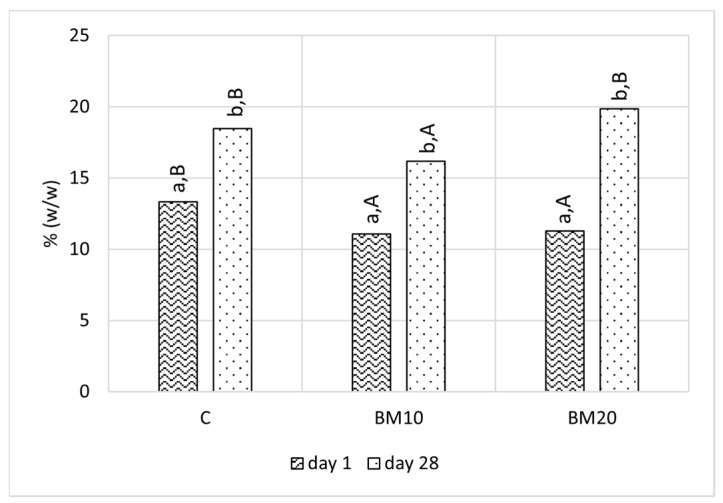
Protein content in Camembert-type cheese at the beginning and end of the ripening period. Explanatory notes: C—control Camembert-type cheese; BM10—Camembert-type cheese with 10% buttermilk; BM20—Camembert-type cheese with 20% of buttermilk. Different small letters (a,b) indicate significant differences in the same cheese on different ripening days. Different capital letters (A,B) indicate significant differences between cheese samples on the same ripening day (*p* ≤ 0.05).

The total protein content was measured at the beginning and end of the ripening period. Directly after production, the protein content ranged from 11.09% to 13.33% and was significantly higher in the control cheese compared to cheese with buttermilk (Figure 4). This may be associated with lower total solid content in samples with BM (Figure 2). During ripening, a notable increase (*p* < 0.05) in the total protein content was observed, and at the end of ripening, it reached values between 16.16% and 19.85%. The increase in protein content, similar to fat, is related to moisture loss in Camembert-type cheese during ripening [50]. Statistical analysis indicated that protein content in BM20 at day 28 did not differ from the control, whereas BM10 had significantly lower (*p* < 0.05) protein content. The protein levels both at the beginning and end of ripening are lower than the results obtained by Los et al. [50], who found 19–22% of protein directly after production and 21–23% of protein after 28 days of ripening Camembert-type cheese samples with different starter cultures. In the study conducted by Bahrami et al. [31] on cheese with sweet BM, samples with 10% and 20% obtained after production did not differ in protein content, which was lower than that of the control, consistent with our findings. In the cited work, the cheese samples were not ripened.

**Figure 5:**
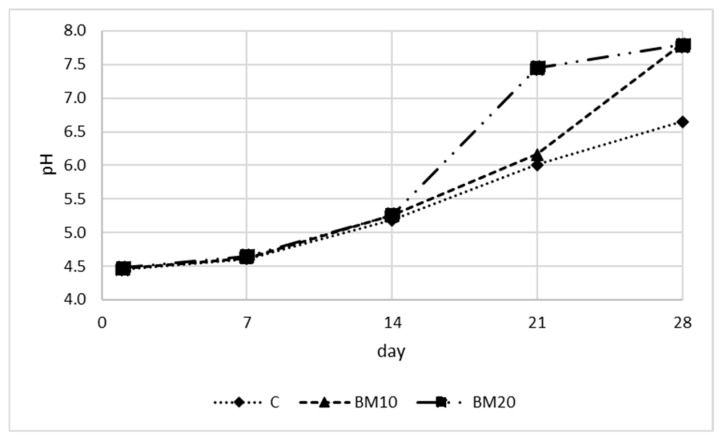
The pH of Camembert-type cheese during the ripening period. Explanatory notes: C—control Camembert-type cheese; BM10—Camembert-type cheese with 10% buttermilk; BM20—Camembert-type cheese with 20% of buttermilk. Standard deviations and results of the HSD Tukey test are presented in the Appendix A.

**Figure 6:**
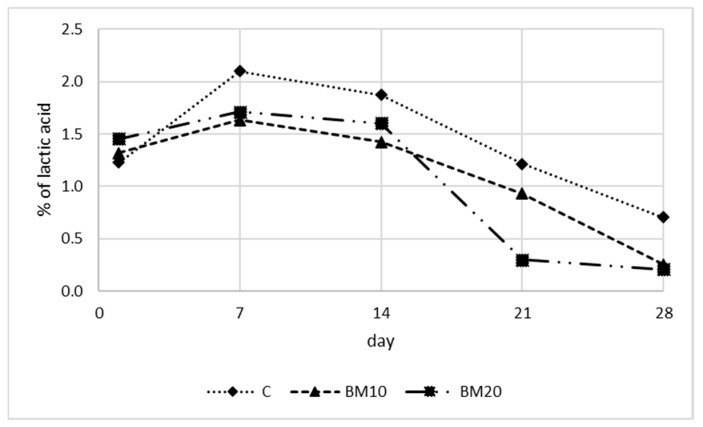
Titratable acidity expressed as % of lactic acid of Camembert-type cheese during the ripening period. Explanatory notes: C—control Camembert-type cheese; BM10—Camembert-type cheese with 10% buttermilk; BM20—Camembert-type cheese with 20% of buttermilk. Standard deviations and results of the HSD Tukey test are presented in the Appendix A.

The changes in acidity during the ripening of Camembert-type cheese are presented in Figure 5 and Figure 6 (pH and titratable acidity expressed as % of lactic acid). The results of the statistical analysis of acidity and water activity are presented in Appendix A (Table A1). Throughout the ripening, a gradual decrease in acidity was observed, which was reflected in increases in pH and a decline in the lactic acid content. The initial pH was 4.45–4.48 and did not differ among cheese variants. During the first two weeks, the pH increased slightly to 4.61, 4.62, and 4.65 on day 7 and 5.18, 5.26, and 5.26 on day 14 for the control, BM10, and BM20, respectively. Subsequently, the increase in pH was more rapid, especially for cheese with buttermilk, reaching 6.01, 6.16, and 7.45 on day 21 and 6.65, 7.81, and 7.79 on day 28, respectively. More distinct pH changes in Camembert-type cheese with BM are probably connected with its higher moisture content and less dense casein gel structure, which facilitate proteolysis and other changes occurring in the cheese during maturation. At the end of ripening, samples BM10 and BM20 did not differ (Table A1). The lack of impact of 10% and 20% buttermilk addition on cheese pH immediately after production was also reported by Bahrami et al. [31].

The initial lactic acid content was 1.23, 1.32, and 1.46, respectively, for the control, BM10, and BM20 cheese. An increase in the lactic acid content was observed between days 1 and 7, which was associated with lactose fermentation by starter bacteria. On day 7, the lactic acid content was 2.10%, 1.64%, and 1.71%, respectively. During the remaining period, lactic acid content decreased dynamically, reaching the lowest values on day 28 (0.71%, 0.26%, and 0.21%, respectively). Similar changes in acidity during the ripening of Camembert-type cheese have also been observed by other authors [7,8,40,47,50].

The reduction in lactic acid is caused by its consumption by mold *P. candidum* developed on the cheese surface during the first two weeks. During weeks 3 and 4, as the mold was already established, the transformation carried out by its enzymes accelerated, resulting in a more dynamic drop in acidity. *P. candidum* metabolizes lactate and results in the proteolysis of cheese proteins, producing ammonia, water, and oxygen [2]. The proteolysis resulting from molds causes a decrease in acidity on the surface of the cheese, and the pH gradient from the surface to the core diminishes over time. The decrease in acidity affects the texture and is responsible for the softening of Camembert-type cheese [8]. As a result of high pH, calcium phosphate precipitates as a layer on the surface of the cheese. This leads to a gradient of calcium phosphate from the cheese core to the surface, with this compound migrating toward the surface. This phenomenon, along with casein proteolysis, contributes to the softening of the cheese interior, which is characteristic of ripened Camembert-type cheese [40]. The high proteolytic activity of molds used in Camembert production results in a cheese with a relatively short ripening period [51].

**Figure 7:**
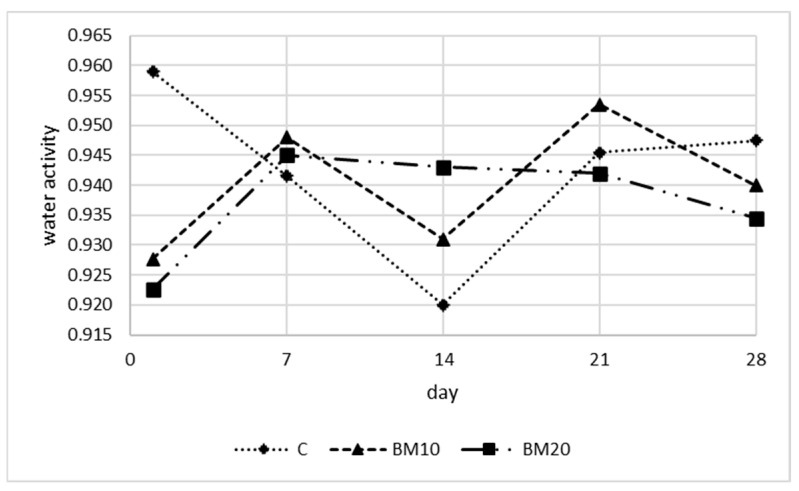
The water activity of Camembert-type cheese during the ripening period. Explanatory notes: C—control Camembert-type cheese; BM10—Camembert-type cheese with 10% buttermilk; BM20—Camembert-type cheese with 20% of buttermilk. Standard deviations and results of the HSD Tukey test are presented in the Appendix A.

The water activity of Camembert-type cheese samples with BM, as well as control Camembert, did not change during the ripening period (Figure 7). Differences among cheese variants were found only on day 1, when the control cheese had significantly higher (*p* < 0.05) a_w_ than BM10 and BM20 (Table A1), with values of 0.959, 0.928, and 0.923, respectively. Los et al. [50] recorded higher values of water activity in Camembert cheese and noted a significant decrease during ripening (from 0.973–0.976 on day 0 to 0.962–0.965 on day 28). High water activity, along with the decline in acidity during ripening, makes Camembert a type of cheese prone to spoilage [2].

### 3.4. Microstructure and Texture

The microstructure of cheese is a crucial factor influencing its quality characteristics and functional properties. Thus, the knowledge about the structure of newly developed products is of high importance for producers. Our study on cheese microstructure enables an understanding of the association between cheese components each other and how they change during ripening [52,53]. Modern methods of microscopy, such as scanning electron microscopy (SEM), transmission electron microscopy (TEM), and confocal laser scanning microscopy (CLSM), provide detailed insights into cheese structure at the microscopic level. Electron microscopy provides images with much higher resolution compared to traditional light microscopy [53,54]. This technique has been applied to analyze the microstructure of various types of cheese, including full-fat Iranian White Cheese [55], Halloumi [56], Cheddar [16], Tallaga cheese [57], and Kashar cheese [58]. SEM has also been used to visualize the microstructure of Camembert cheese. Michalski et al. [59] analyzed the SEM images of Camembert with different fat globule sizes. In the case of cheese, the SEM method clearly illustrates how cheese components like protein, fat, water channels, microorganisms, and precipitated minerals interact [52,53]. The SEM method was also used in studies on other dairy products like buttermilk [60,61], yogurt, and other fermented milk and powdered milk products [62]. Besides dairy products, SEM was used to observe the microstructure of meat and soup [63].

**Figure 8:**
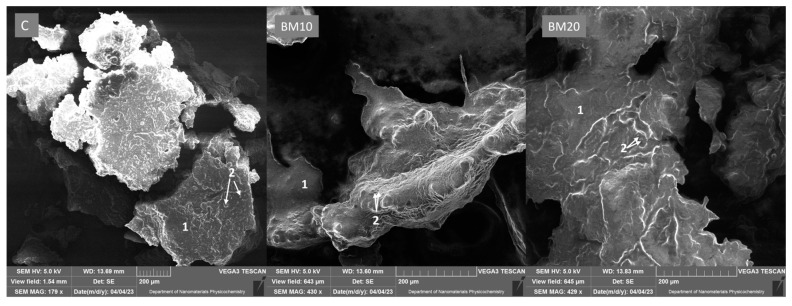
SEM images of ripened Camembert-type cheese microstructure. Explanatory notes: C—control Camembert-type cheese; BM10—Camembert-type cheese with 10% buttermilk; BM20—Camembert-type cheese with 20% of buttermilk; 1—protein matrix; 2—fat globules.

In terms of microstructure, cheese is a complex material composed of a protein matrix made from coagulated casein micelles to which other components such as fat, water, minerals, and microorganisms are bound. The arrangement of the microstructure is affected by several factors, including the type of raw material, cheese type, processing parameters, the addition of calcium chloride, and the type of microbiological culture used for ripening. The microstructure impacts the cheese’s texture and quality characteristics [53,64,65]. Each type of cheese has characteristic microstructural properties, which are related to chemical and biological transformations [55]. The microstructure of Camembert-type cheese samples observed on the 21st day of ripening is presented in Figure 8.

The images of the control cheese are coarser and more porous compared to cheese samples with 10% and 20% buttermilk. However, the images of BM10, BM20, and the control cheese show considerable similarity, indicating that the level of buttermilk addition does not notably alter the structural characteristics of the products. The microstructure of all samples is characterized by a compact and dense structure. The surface of the protein matrix in BM10 and BM20 samples is very even, homogenous, and free from ripples, which may be connected with the smooth and soft texture of Camembert-type cheese at this stage of ripening. Krebs et al. [48] observed the microstructure of rennet cheese produced from ultrafiltered buttermilk or skim milk. The results of SEM visualization showed a more irregular cheese matrix microstructure for buttermilk cheese compared to milk cheese, which contrasts with our findings and may be related to differences in cheese type and maturity level. The compact structure of the protein matrix on the 21st day of ripening was also observed by Rahimi et al. [55] in full-fat Iranian White Cheese. Romeih et al. [16] analyzed the microstructure of low-fat Cheddar cheese with the addition of buttermilk powder or skim milk powder. The author revealed that buttermilk addition resulted in a more homogenous and denser microstructure observed with the SEM technique, which is in line with our findings. This is explained by the ability of MFGM present in buttermilk to form cross-links with the casein matrix. This interaction strongly affects the microstructure, texture, and water-holding capacity of cheese [13,16,48]. The fat globules in the cheese microstructure are sparse in the field of view. This indicates that fat is present not only in the form of emulsified fat globules but also largely in the form of free oil pools, which are caused by the coalescence of fat globules, probably linked to the high degree of cheese maturity. During cheese ripening, fat globules aggregate and coalesce, forming large fat particles and pools of free oil. This phenomenon is accelerated by the changes in the structure of the casein matrix, which stabilizes fat globules. During ripening, the degradation of the casein matrix, in which fat globules are embedded, occurs. Residual chymosin, indigenous milk enzymes, and proteases from starter culture microorganisms facilitate the proteolysis of casein. Moreover, the calcium bound to the casein solubilizes over time. As a result, the cheese softens during the ripening process [52,55,66].

The instrumental texture analysis of cheese enables the characterization of various mechanical properties that reflect the product’s structure, mouthfeel, and acceptability. Several factors affect cheese texture, including moisture; pH; fat content; protein content; and notably, the structure of the protein matrix [23]. In this study, the analysis of ripened (day 28) Camembert-type cheese allowed for the determination of the following texture parameters: hardness, adhesiveness, springiness, cohesiveness, gumminess, chewiness, and resilience. Both cheese samples with BM addition exhibited significantly lower (*p* < 0.05) hardness, adhesiveness, and gumminess than the control. No significant differences were observed for the other parameters. Additionally, variants BM10 and BM20 differed only in terms of adhesiveness, with the cheese containing a higher BM content and therefore being more adhesive (Table 3).

Camembert is a type of soft cheese, and its hardness decreases during ripening. This is due to the intense proteolysis resulting from molds. The hydrolysis of the cheese protein network into small peptides and free amino acids leads to the soft texture of the cheese. Additionally, the high water and fat contents contribute to the softness of Camembert [2,40,55]. In the study by Los et al. [50], the hardness value measured on day 28 ranged from 1 to 14 N; our results fall within this range. Low hardness indicates that the ripening process has proceeded properly.

According to Bielska and Cais-Sokolińska [39], the texture as well as the sensory properties can be improved by BM addition. Bahrami et al. [31], who produced cream cheese from milk combined with sweet BM, found that the cheese with BM had a softer body, which is consistent with our study. The authors suggested that this effect is connected to changes in the electric charge of casein during cream churning and the presence of milk fat globule membrane (MFGM) components, mainly phospholipids (PLs) and free fat in the BM. The lower hardness of cheese from ultrafiltered BM compared with cheese from skim milk was also stated by Krebs et al. [48]. Romeih et al. [16] found lower hardness in Cheddar cheese with MFGM components.

### 3.5. Color and Sensory Properties

The color and appearance of cheese are important factors in consumer acceptability and directly influence the quality of the product. Color is the first property of cheese noticed by consumers and may determine whether it will be accepted or rejected [23,43]. The color values of the tested cheese variants were significantly affected by the ripening time and BM addition (Table 4). During ripening, L* and WI values both in the paste and on the surface of Camembert-type cheese samples with BM decreased, with the exception of WI on the surface of BM10, in which these parameters remained stable. However, at the end of ripening, BM cheese samples did not differ from the control with respect to these parameters. In regard to all cheese variants, values of a* and b* parameters increased in the interior and were stable in the exterior. BM cheese samples were characterized by lower a* values in the interior and higher a* values in the exterior compared to the control. Additionally, BM10 and BM20 had lower b* values in the cheese paste. The stability of color parameters on the cheese rind indicates a full and even coating with mycelium. The similarity of both BM Camembert-type cheese samples to the control was particularly evident at the end of the ripening period with regard to lightness and the whiteness index. This similarity suggests that the color of the developed cheese products will be appealing to consumers.

Camembert-type cheese made without or with 10% and 20% of BM addition underwent sensory assessment throughout the entire ripening period (Table 5). For appearance, consistency, and aroma, the grades were high (from rather good to very good) during the entire ripening process, and there were no statistically significant differences among cheese samples. Also, the overall acceptability did not differ. However, the taste of Camembert-type cheese samples with BM was significantly lower (*p* < 0.05) than the control at the end of the ripening period. Presumably due to the higher moisture content in cheese samples with BM, the process of ripening was faster, and the most attractive properties were reached already after 2 weeks of ripening.

The changes in the flavor of Camembert during the ripening period result from the enzymatic activity of rennet, lactic acid bacteria (LAB) present in the starter culture, and the mycelium. The aroma of ripened Camembert cheese is shaped by ammonia and other compounds produced from amino acids through processes such as deamination, decarboxylation, and further transformation. Volatile sulfur compounds also play a role in forming the characteristic Camembert flavor. Dimethyl sulfide and dimethyl trisulfide contribute to the garlicky flavor of a matured Camembert [2,67]. Moreover, at the later stages of ripening, the increasing concentration of bitter compounds—specifically hydrophobic peptides released during proteolysis—may negatively impact the taste of the cheese [50]. Consequently, an extended ripening period for Camembert-type cheese may diminish its appeal. It is advisable to shorten the ripening period of Camembert-type cheese with BM addition, which is economically beneficial for the cheesemaking industry. For better insights into the sensory quality of BM Camembert-type cheeses, and to predict their commercial success, it is reasonable to perform a consumer acceptability test.

## 4. Conclusions

The Camembert-type cheese samples with 10% and 20% buttermilk addition to milk exhibited differences in the production yield; composition; acidity; and microstructure, texture, and color compared to the control. The tested cheese samples did not show significant differences in sensory attributes. When comparing the variants with 10% and 20% BM, cheese BM20 demonstrated a higher production yield and higher protein content at the end of the ripening period compared to BM10. Therefore, a 20% BM addition can be considered optimal, as it is also economically favorable by allowing for the management of a greater amount of by-product. Furthermore, incorporating BM into Camembert-type cheese may enhance its nutritional value due to the increased concentration of phospholipids (PL). In conclusion, sweet BM could serve as a partial substitute for milk in Camembert-type cheese manufacturing. The partial substitution of milk with BM may be economically beneficial for the dairy industry, as it helps manage a by-product of butter production and reduces the production cost of Camembert-type cheese.

## Figures and Tables

**Table 1:** The operating conditions of TPA.

Measurement	Condition
Probe	stainless steel cylindrical rod (4 mm diameter)
Pre-test speed	5.0 mm/s
Test speed	1.0 mm/s
Post-test speed	5.0 mm/s
Distance	20.0 mm
Time	5.00 s
Strain	70%
Force	Grams
Trigger force	5.0 g

**Table 2:** Physicochemical properties and color of milk and buttermilk.

Parameter	Milk	Buttermilk	ANOVA	
			F	*p*
physicochemical parameters				
pH	6.69 ± 0.01	6.75 ± 0.01	51.200	0.0020 *
% of l.a.	0.16 ± 0.01	0.15 ± 0.01	4.500	0.1012
fat, % (*w*/*w*)	3.23 ± 0.06	1.37 ± 0.06	1568.000	0.0000 *
protein, % (*w*/*w*)	3.50 ± 0.02	3.85 ± 0.03	272.898	0.0001 *
total solids, % (*w*/*w*)	11.89 ± 0.07	10.15 ± 0.07	946.125	0.0000 *
color parameters				
L*	76.80 ± 0.22	72.41 ± 0.26	837.624	0.0000 *
a*	−2.28 ± 0.05	−3.72 ± 0.06	1883.708	0.0000 *
b*	3.81 ± 0.16	2.42 ± 0.20	153.341	0.0000 *
whiteness index	76.38 ± 0.19	72.05 ± 0.23	1039.919	0.0000 *

Explanatory notes: Table presents mean values ± standard deviation. l.a.—lactic acid; * statistically significant differences (*p* ≤ 0.05).

**Table 3:** Texture parameters of Camembert-type cheese at the end of the ripening period.

Texture Parameters	C	BM10	BM20	ANOVA	
				F	*p*
Hardness, N	6.887 ^b^ ± 0.595	4.425 ^a^ ± 0.463	4.902 ^a^ ± 0.280	39.8735	0.0000 *
Adhesiveness, g·sec	−2489.34 ^c^ ± 348.34	−521.50 ^a^ ± 276.21	−688.62 ^b^ ± 131.22	82.6731	0.0000 *
Springiness	0.940 ± 0.057	0.884 ± 0.114	0.940 ± 0.029	1.0765	0.3657
Cohesiveness	0.272 ± 0.100	0.291 ± 0.088	0.266 ± 0.046	0.1555	0.8573
Gumminess	1.679 ^b^ ± 0.195	1.280 ^a^ ± 0.389	1.194 ^a^ ± 0.078	3.9163	0.0491 *
Chewiness	1.607 ± 0.151	1.158 ± 0.467	1.231 ± 0.265	2.2681	0.1429
Resilience	0.012 ± 0.003	0.017 ± 0.011	0.016 ± 0.002	0.9418	0.4118

Explanatory notes: Table presents mean values ± standard deviation. C—control Camembert-type cheese; BM10—Camembert-type cheese with 10% buttermilk; BM20—Camembert-type cheese with 20% of buttermilk; * statistically significant differences (*p* ≤ 0.05). Different letters (^a–c^) indicate significant differences between the mean values in the same row.

**Table 4:** Color parameters of Camembert-type cheese during ripening.

Ripening Day	C	BM10	BM20
L* interior			
1	86.33 ^a,A^ ± 1.86	95.86 ^c,B^ ± 1.41	95.71 ^c,B^ ± 0.37
7	87.65 ^a,b,A^ ± 2.06	94.63 ^b,c,B^ ± 1.16	93.86 ^b,c,B^ ± 0.45
14	89.80 ^a,b,A^ ± 0.29	93.26 ^b,B^ ± 0.68	91.94 ^b,B^ ± 1.58
21	90.55 ^b,A^ ± 1.61	87.32 ^a,B^ ± 1.02	88.22 ^a,B^ ± 1.23
28	88.05 ^a,b^ ± 3.04	85.16 ^a^ ± 0.94	88.25 ^a^ ± 1.55
a* interior			
1	−1.70 ^a,B^ ± 0.13	−2.34 ^a,A^ ± 0.13	−2.35 ^a,A^ ± 0.04
7	−1.51 ^b,B^ ± 0.02	−2.26 ^a,A^ ± 0.09	−2.28 ^a,A^ ± 0.04
14	−1.38 ^b,B^ ± 0.05	−1.71 ^b,A^ ± 0.13	−1.64 ^c,A^ ± 0.06
21	−1.40 ^b,B^ ± 0.05	−1.15 ^c,C^ ± 0.09	−1.94 ^b,A^ ± 0.12
28	−1.50 ^b,A^ ± 0.06	−1.10 ^c,B^ ± 0.12	−1.07 ^d,B^ ± 0.14
b* interior			
1	10.53 ^a,B^ ± 0.45	9.13 ^a,A^ ± 0.05	9.26 ^a,A^ ± 0.12
7	12.33 ^b,C^ ± 0.22	10.55 ^b,A^ ± 0.02	11.14 ^c,B^ ± 0.23
14	13.92 ^d,C^ ± 0.02	11.42 ^c,A^ ± 0.36	11.96 ^d,B^ ± 0.23
21	13.09 ^c,C^ ± 0.35	11.73 ^c,B^ ± 0.29	10.11 ^b,A^ ± 0.04
28	12.32 ^b,B^ ± 0.59	10.84 ^b,A^ ± 0.26	11.51 ^c,A^ ± 0.28
whiteness index interior			
1	82.61 ^A^ ± 1.20	89.64 ^d,B^ ± 0.59	89.53 ^d,B^ ± 0.23
7	82.44 ^A^ ± 1.44	87.91 ^c,B^ ± 0.44	87.07 ^c,B^ ± 0.37
14	82.68 ^A^ ± 0.19	86.62 ^b,C^ ± 0.39	85.44 ^b,B^ ± 0.92
21	83.74 ^A,B^ ± 0.88	82.68 ^a,A^ ± 0.75	84.34 ^a,b,B^ ± 0.94
28	82.64 ± 1.95	81.62 ^a^ ± 0.73	83.49 ^a^ ± 1.17
L* exterior			
14	95.08 ^B^ ± 1.21	93.45 ^b,A,B^ ± 0.60	92.87 ^b,A^ ± 1.69
21	94.59 ^B^ ± 1.27	92.31 ^a,b,A,B^ ± 1.00	89.46 ^a,b,A^ ± 2.98
28	91.40 ± 3.38	91.37 ^a^ ± 1.24	88.16 ^a^ ± 1.96
a* exterior			
14	−0.80 ^B^ ± 0.11	−0.33 ^A^ ± 0.04	−0.44 ^A^ ± 0.13
21	−0.73 ^C^ ± 0.15	−0.17 ^A^ ± 0.12	−0.40 ^B^ ± 0.10
28	−0.68 ^B^ ± 0.08	−0.22 ^A^ ± 0.12	−0.35 ^A^ ± 0.22
b* exterior			
14	7.08 ± 0.78	7.27 ± 0.15	7.56 ± 1.15
21	7.82 ± 0.63	8.17 ± 1.87	7.43 ± 0.72
28	7.17 ± 1.22	6.61 ± 0.61	8.16 ± 1.51
whiteness index exterior			
14	91.28 ^B^ ± 0.89	90.20 ^A,B^ ± 0.50	89.51 ^b,A^ ± 1.36
21	90.41 ^B^ ± 0.84	88.75 ^A,B^ ± 1.95	86.93 ^a,b,A^ ± 1.98
28	88.63 ± 2.93	89.12 ± 1.32	85.56 ^a^ ± 1.97

Explanatory notes: Table presents mean values ± standard deviation. C—control Camembert-type cheese; BM10—Camembert-type cheese with 10% buttermilk; BM20—Camembert-type cheese with 20% of buttermilk. Different superscript letters (^a–d^) indicate significant differences in the same cheese on different ripening days. Superscript capital letters (^A–C^) indicate significant differences between cheese samples on the same ripening day (*p* ≤ 0.05).

**Table 5:** Sensory assessment of Camembert-type cheese during ripening.

Ripening Day	C	BM10	BM20
appearance			
1	6.75 ± 0.50	6.13 ± 0.85	6.25 ± 0.96
7	6.00 ± 0.82	6.67 ± 0.58	6.67 ± 0.58
14	6.50 ± 1.00	7.00 ± 0.00	7.00 ± 0.00
21	6.00 ± 0.82	5.67 ± 1.15	6.00 ± 1.73
28	6.50 ± 1.00	5.75 ± 1.89	5.50 ± 1.73
consistency			
1	5.88 ± 0.63	5.50 ± 1.00	5.63 ± 1.11
7	5.25 ± 0.96	6.00 ± 1.00	6.00 ± 1.00
14	5.25 ± 1.71	7.00 ± 0.00	6.67 ± 0.58
21	4.25 ± 1.71	6.33 ± 1.15	4.33 ± 0.58
28	5.38 ± 0.48	6.25 ± 1.50	6.00 ± 1.41
taste			
1	5.25 ± 1.26	6.25 ^b^ ± 0.50	6.75 ^c^ ± 0.50
7	6.25 ± 0.50	5.33 ^a,b^ ± 0.58	6.67 ^c^ ± 0.58
14	5.00 ± 1.41	5.67 ^a,b^ ± 1.53	6.33 ^b,c^ ± 0.58
21	5.00 ± 1.83	5.67 ^a,b^ ± 1.53	3.50 ^a,b^ ± 1.80
28	5.88 ± 0.85	3.75 ^a^ ± 0.96	3.38 ^a^ ± 1.49
aroma			
1	6.00 ± 1.15	6.38 ± 0.48	6.25 ± 0.96
7	6.00 ± 0.82	5.67 ± 1.15	6.33 ± 0.58
14	4.75 ± 1.50	6.00 ± 1.00	6.00 ± 1.00
21	4.25 ± 0.96	4.67 ± 0.58	5.50 ± 0.71
28	5.50 ± 0.58	5.13 ± 1.65	4.75 ± 1.89
overall acceptability			
1	5.50 ± 0.58	5.63 ± 0.48	5.88 ± 0.63
7	6.00 ± 0.82	6.17 ± 0.29	6.33 ± 0.58
14	5.63 ± 0.75	6.00 ± 0.87	6.17 ± 0.29
21	5.50 ± 1.29	5.83 ± 1.15	4.00 ± 1.50
28	5.88 ± 0.63	4.38 ± 1.97	4.25 ± 1.94

Explanatory notes: Table presents mean values ± standard deviation. C—control Camembert-type cheese; BM10—Camembert-type cheese with 10% buttermilk; BM20—Camembert-type cheese with 20% of buttermilk. Different superscript letters (^a–c^) indicate significant differences in the same cheese on different ripening days.

## Data Availability

The original contributions presented in the study are included in the article, further inquiries can be directed to the corresponding author.

## Appendix A

The results of the HSD Tukey test performed for pH, lactic acid content, and water activity.

**Table A1:** pH, % of lactic acid, and water activity of Camembert-type cheese during ripening.

Ripening Day	C	BM10	BM20
pH			
1	4.45 ± 0.01 ^a^	4.46 ± 0.01 ^a^	4.48 ± 0.02 ^a^
7	4.61 ± 0.01 ^a,A^	4.62 ± 0.01 ^b,A^	4.65 ± 0.00 ^b,B^
14	5.18 ± 0.04 ^b,A^	5.26 ± 0.10 ^c,A^	5.26 ± 0.08 ^c,A^
21	6.01 ± 0.04 ^c,A^	6.16 ± 0.03 ^d,A^	7.45 ± 0.21 ^d,B^
28	6.65 ± 0.23 ^d,A^	7.81 ± 0.03 ^e,B^	7.79 ± 0.04 ^e,B^
% of lactic acid			
1	1.23 ± 0.17 ^b^	1.32 ± 0.03 ^c^	1.46 ± 0.03 ^b^
7	2.10 ± 0.13 ^c,B^	1.64 ± 0.03 ^d,A^	1.71 ± 0.00 ^b,A^
14	1.88 ± 0.07 ^c,B^	1.43 ± 0.14 ^c,A^	1.61 ± 0.20 ^b,A,B^
21	1.22 ± 0.12 ^b,C^	0.93 ± 0.03 ^b,B^	0.30 ± 0.05 ^a,A^
28	0.71 ± 0.07 ^a,B^	0.26 ± 0.05 ^a,A^	0.21 ± 0.03 ^a,A^
water activity (a_w_)			
1	0.959 ± 0.021 ^B^	0.928 ± 0.003 ^A,B^	0.923 ± 0.003 ^A^
7	0.942 ± 0.021	0.948 ± 0.020	0.945 ± 0.020
14	0.920 ± 0.004	0.931 ± 0.018	0.943 ± 0.021
21	0.946 ± 0.022	0.954 ± 0.022	0.942 ± 0.020
28	0.948 ± 0.023	0.940 ± 0.023	0.935 ± 0.022

Explanatory notes: Table presents mean values ± standard deviation. C—control Camembert-type cheese; BM10—Camembert-type cheese with 10% buttermilk; BM20—Camembert-type cheese with 20% of buttermilk. Different superscript letters (^a–e^) indicate significant differences in the same cheese on different ripening days. Superscript capital letters (^A–C^) indicate significant differences between cheese samples on the same ripening day (*p* ≤ 0.05).
